# Survival Prediction Analysis in Glioblastoma With Diffusion Kurtosis Imaging

**DOI:** 10.3389/fonc.2021.690036

**Published:** 2021-07-14

**Authors:** Yuan Li, Michelle M. Kim, Daniel R. Wahl, Theodore S. Lawrence, Hemant Parmar, Yue Cao

**Affiliations:** ^1^ Departments of Radiation Oncology, University of Michigan, Ann Arbor, MI, United States; ^2^ Department of Biomedical Engineering, University of Michigan, Ann Arbor, MI, United States; ^3^ Department of Radiology, University of Michigan, Ann Arbor, MI, United States

**Keywords:** diffusion kurtosis imaging, diffusion MRI, glioblastoma, survival prediction, imaging analysis

## Abstract

**Simple Summary:**

Glioblastoma (GBM) is the most common and aggressive primary brain tumor. Diffusion kurtosis imaging (DKI) has characterized non-Gaussian diffusion behaviors in brain normal tissue and gliomas, but there are very limited efforts in investigating treatment responses of kurtosis in GBM. This study aimed to investigate whether any parameter derived from the DKI is a significant predictor of overall survival (OS). We found that the large mean, 80 and 90 percentile kurtosis values in the contrast enhanced gross tumor volume (Gd-GTV) on post-Gd T1-weighted images pre-RT were significantly associated with reduced OS. In the multivariate Cox model, the mean kurtosis Gd-GTV pre-RT after considering effects of age, extent of surgery, and methylation were significant predictors of OS. In addition, the 80 and 90 percentile kurtosis values in Gd-GTV post RT were significantly associated with progression free survival (PFS). The DKI model demonstrates the potential to predict outcomes in the patients with GBM.

**Purpose:**

Non-Gaussian diffusion behaviors in gliomas have been characterized by diffusion kurtosis imaging (DKI). But there are very limited efforts in investigating the kurtosis in glioblastoma (GBM) and its prognostic and predictive values. This study aimed to investigate whether any of the diffusion kurtosis parameters derived from DKI is a significant predictor of overall survival.

**Methods and Materials:**

Thirty-three patients with GBM had pre-radiation therapy (RT) and mid-RT diffusion weighted (DW) images. Kurtosis and diffusion coefficient (DC) values in the contrast enhanced gross tumor volume (Gd-GTV) on post-Gd T1 weighted images pre-RT and mid-RT were calculated. Univariate and multivariate Cox models were used to evaluate the DKI parameters and clinical factors for prediction of OS and PFS.

**Results:**

The large mean kurtosis values in the Gd-GTV pre-RT were significantly associated with reduced OS (p = 0.02), but the values at mid-RT were not (p > 0.8). In the multivariate Cox model, the mean kurtosis in the Gd-GTV pre-RT (p = 0.009) was still a significant predictor of OS after adjusting effects of age, O6-Methylguanine-DNA Methyl transferase (MGMT) methylation and extent of resection. In Gd-GTV post-RT, 80 and 90 percentile kurtosis values were significant predictors (p ≤ 0.05) for progression free survival (PFS).

**Conclusion:**

The DKI model demonstrates the potential to predict OS and PFS in the patients with GBM. Further development and histopathological validation of the DKI model will warrant its role in clinical management of GBM.

## Introduction

Glioblastoma (GBM) is the most common and aggressive primary brain tumor in adults and has a poor prognosis with a median survival of approximately 14 months despite multimodality therapy with surgery, concurrent chemoradiation therapy, and adjuvant chemotherapy (1, 2). Standard clinical assessment of tumor progression or therapy response (3) is based primarily on post-contrast T1-weighted and fluid-attenuated inversion recovery (FLAIR) T2-weighted magnetic resonance images (MRI). There are some challenges to these conventional techniques. The contrast enhancement on the post-contrast T1-weighted MRI is affected by tumor growth, but also radiation, anti-angiogenesis drugs, and chemotherapy, all of which can be attributed to blood–brain barrier disruption. Abnormality on T2 FLAIR images is influenced by T2 changes of tumor cells as well as by edema that co-exists within GBM or is affected by radiation therapy. Limitations of conventional MRI in clinical management of GBM have motivated investigations of physiological and metabolic MRI.

Diffusion weighted (DW) imaging has been proposed to overcome these limitations. DW imaging is a technique to measure water molecule mobility in the microscopic tissue environment and is sensitive to cell density and size, cell membrane permeability, and extracellular space tortuosity. Apparent diffusion coefficient (ADC) quantified from conventional DW images fitted to a mono-exponential function is the commonly reported parameter in literature. The correlation between high cellularity and low ADC in tumor animal models and human cancers motivates investigations on roles of ADC in clinical GBM (4–7). However, heterogeneous tissue in GBM, especially edema, often results in elevated ADC compared to normal white matter (WM) (1) and gray matter (GM). To overcome this limitation, high b-value DW images and high-order diffusion models have been explored in clinical gliomas to differentiate tumor grade and assess therapy response (8–17). Diffusion tensor imaging (DTI) is an emerging technique to investigate brain tumor. Fractional anisotropy (FA) that derived from DTI has been suggested to provide information of cell density. A previous study of FA in GBM showed that FA was low in GBM and suggested that the directional diffusion has been corrupted in the tumor region (18). However, how to differentiate low FA caused by tumor from that affected by edema is a challenge.

The signal-to-noise ratio of diffusion weighted images acquired on clinical scanners is a limiting factor in the application of high-order diffusion models to GBM. Diffusion kurtosis imaging (DKI) is an emerging approach to estimate the non-Gaussian water diffusion behavior over high b values in tissue. DKI has shown the potential to characterize normal and pathologic tissue (17, 19). Previous research has suggested that DKI provides better separation of brain tumor grades (14, 17, 20), but there are very limited efforts in investigating treatment responses of kurtosis in GBM and its prognostic and predictive values for patient survival (21).

In this study, we hypothesized that high diffusion kurtosis in GBM correlated with decreased OS. We applied the diffusion kurtosis model to the DW images acquired in the patients with GBM before radiation therapy (pre-RT), during the course of RT (mid-RT) and after radiation therapy (post-RT). We analyzed the parameter differences between pre-RT and mid-RT to investigate the bio-physical meaning of the parameters and response to RT. Finally, we tested whether any parameter derived from the model is a significant predictor of overall survival (OS).

## Materials and Methods

### Patients

Thirty-three patients with histologically confirmed, newly diagnosed GBM were enrolled on prospective, institute-review-board approved protocols. All patients signed written informed consent. The patients had research MRI scans, including anatomic scans and diffusion weighted (DW) images, pre-RT following maximal tumor surgical resection prior to chemo-radiation therapy (CRT) and during the 3rd–4th week of CRT (mid-RT). The twenty-one patients had the research MRI scans 3-month post-RT. The ten patients were treated based upon the institution protocol of concurrent CRT following chemotherapy with a median dose of 60 Gy (40.05–72 Gy), and the 23 patients were enrolled on a prospective radiation boosting clinical trial and treated to 75 Gy (NCT02805179) (22). All patients received concurrent temozolomide.

### *In Vivo* MR imaging

All MRI scans were performed on a 3.0-T scanner (Skyra, Siemens Healthineers) using a 20-channel head coil. Conventional MR images, 2D T2-FLAIR images, and 3D pre- and post-contrast T1-weighted images using a MPRAGE sequence, were acquired. DW images were acquired by a spin-echo echo-planar pulse sequence with diffusion weighting in three orthogonal directions and 11 b-values from 0 to 2,500 s/mm^2^ with an incremental step of 250 s/mm^2^. Other acquisition parameters included a parallel imaging factor of 4 (GRAPPA) (to reduce echo spacing and hence geometric distortion), TE/TR = 93/9,300 ms, bandwidth of 1,040 Hz/pixel, voxel size of approximately 1.3 × 1.3 × 5.2 mm, 30 slices to cover the whole brain, one average and total scan time of 4.50 min. All DW images were acquired prior to contrast injection.

### Diffusion Model

The diffusion kurtosis model analyzes non‐Gaussian water diffusivity with equation:

(1) $$S=S_{0}*e^{\left(-b*D+\frac{1}{6}{\left(b*D\right)}^{2}*K\right)}$$

where *S*_0_ is an amplitude of diffusion signals, *D* is a diffusion coefficient (DC) that is corrected for the observed non‐Gaussian diffusion behavior and *K* represents an apparent diffusional kurtosis. Here, we did not consider an anisotropic diffusion kurtosis in GBM due to the low anisotropic diffusion behavior in the contrast-enhanced tumor volume.

### Computation of Kurtosis and DC Maps

Kurtosis and DC maps were generated from DW images with 11 b-values using in-house Functional Image Analysis Tools (imFIAT). We first took a logarithm of diffusion signals, and then used Simplex algorithm to fit the model. In the computation process, a 2D 3 × 3 Gaussian filter and brain mask were first applied to all phases of diffusion weighted images to reduce noise influence on the parameter maps.

The gadolinium enhancement gross tumor volumes (Gd-GTV) on post-Gd T1 weighted images were delineated by radiation oncologists who treated the patients. Surgical cavities were removed from the Gd-GTV. The median of the residual Gd-GTV is 20.97 cm^3^ (ranges from 2.33 to 62.50 cm^3^). In eighteen patients with gross total resection (**Table 1**), the median of the residual Gd-GTV (excluding the surgical cavity) was 14.00 cm^3^ (ranges from 2.33 to 46.00 cm^3^).

**Table 1:** Patients characteristics.

Count	N
Patients	33
**Age**	
Median (IQR)	61 (50, 79)
**Gender**	
Female	13 (39.4%)
Male	20 (60.6%)
**ECOG**	
0	7 (21.2%)
1	23 (69.7%)
2	3 (9.1%)
**Median physical dose**	
Institute protocol	60 (40.05, 72)
Boosting protocol	75 (75, 75)
**Extent of surgery**	
Biopsy	6 (18.2%)
Subtotal resection	9 (27.3%)
Gross total resection	18 (54.5%)
**MGMT methylation**	
Positive	9 (27.2%)
Negative	22 (66.7%)
Unknown	2 (6.1%)
**IDH status**	
Mutant	1 (3%)
Wild type	31 (94%)
Unknown	1 (3%)

Considering GBM is a heterogeneous tumor with edema (possible low cellular density) and high cellular components, a mean value of kurtosis or diffusion coefficient averaged over the whole volume of Gd-GTV-cavity could wash out the component that could be more aggressive and predict outcomes. Therefore, we attempted to analyze the part of the histogram of kurtosis or diffusion coefficient, which is associated with the aggressive tumor. Since high kurtosis values and low diffusion coefficients are associated with tumor aggressiveness, we choose high percentiles of kurtosis and low percentiles of DC to test whether they predicted OS. Therefore, mean, 80 and 90 percentile values of kurtosis, and mean, 10 and 20 percentile values of DC in the Gd-GTV pre-RT and mid-RT were calculated.

### Statistical Analysis

The primary endpoint of the study was to determine whether the DKI parameters provide additional predictive values over clinical variables for OS. OS was defined as the interval from the start of RT to death from any cause. Patients were censored at the time of last contact or clinical follow-up, whichever occurred last. Patients were generally followed every 8 weeks after chemoradiation with clinical exam and MRI. Progression-free survival (PFS) was defined as the interval from the start of RT to progression or death, whichever occurred first, and patients were censored at the time of last imaging follow-up. Progression was determined by a multidisciplinary tumor board, and worsened enhancement within 3 months of chemoradiation was generally managed by repeat imaging to rule out pseudoprogression. Progression was defined as worsened enhancement outside of the radiation field, or within the radiation field if progression was confirmed pathologically or with serial confirmatory imaging and clinical evaluation, or by change in therapy (*i.e.* initiation of next-line chemotherapy), whichever occurred first.

PFS and OS were calculated using Kaplan–Meier method. To test predictive values of the DKI parameters, univariate Cox proportional hazards model first was used to evaluate each of the DKI parameters as well as clinical factors for prediction of OS and PFS.

Clinical factors included age (continuous), sex, ECOG performance status (0 *vs*. 1 *vs.* 2), radiation dose (continuous), extent of resection (EOR, gross total resection = 2, subtotal resection = 1, or biopsy = 0), MGMT methylation status (methylated *vs*. unmethylated), and baseline contrast enhanced gross tumor volume (GTV-Gd). Multivariate Cox proportional hazards model was further performed to test whether the DKI parameters could provide additional values to clinical factors for prediction of OS and PFS, adjusting age, MGMT methylation status and EOR. The changes in the DKI parameters at mid-RT compared to pre-RT were also tested using a paired t test. A P-value <0.05 was considered significant.

## Results

### Patient Characteristics and Outcomes

Thirty-three patients who had newly diagnosed GBM treated between October 2012 and December 2018 and had the diffusion imaging scans pre-RT, mid-RT and post-RT as described in the section *In Vivo MR Imaging* were included in this analysis. The patient characteristics are provided in **Table 1**. The median age was 61 years old (50–79). Thirteen patients were female. ECOG performance status of thirty patients was 0–1. Eighteen patients had total surgical resection, nine had subtotal resection and six had biopsy only. Eight of the 31 patients who had MGMT methylation tests were methylated, and one of the 32 patients who had IDH tests had the mutated type.

**Figure 1:**
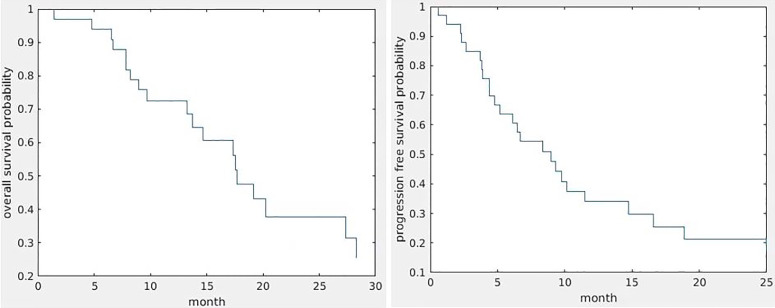
Kaplan–Meier curves of OS (left) and PFS (right).

Fourteen patients were still alive with a median follow-up of 17.4 months (9.07–49.4 months). The median survival was 13.7 months (0.6–37.5 months). Twenty-five patients progressed with a median progression of 8 months (0.6–25 months); one patient progressed (3 weeks) at mid-RT. **Figure 1** shows Kaplan–Meier curves of OS and PFS.

### Kurtosis and DC Values in the Gd-GTV Pre-RT, Mid-RT and Post-RT

**Figure 2:**
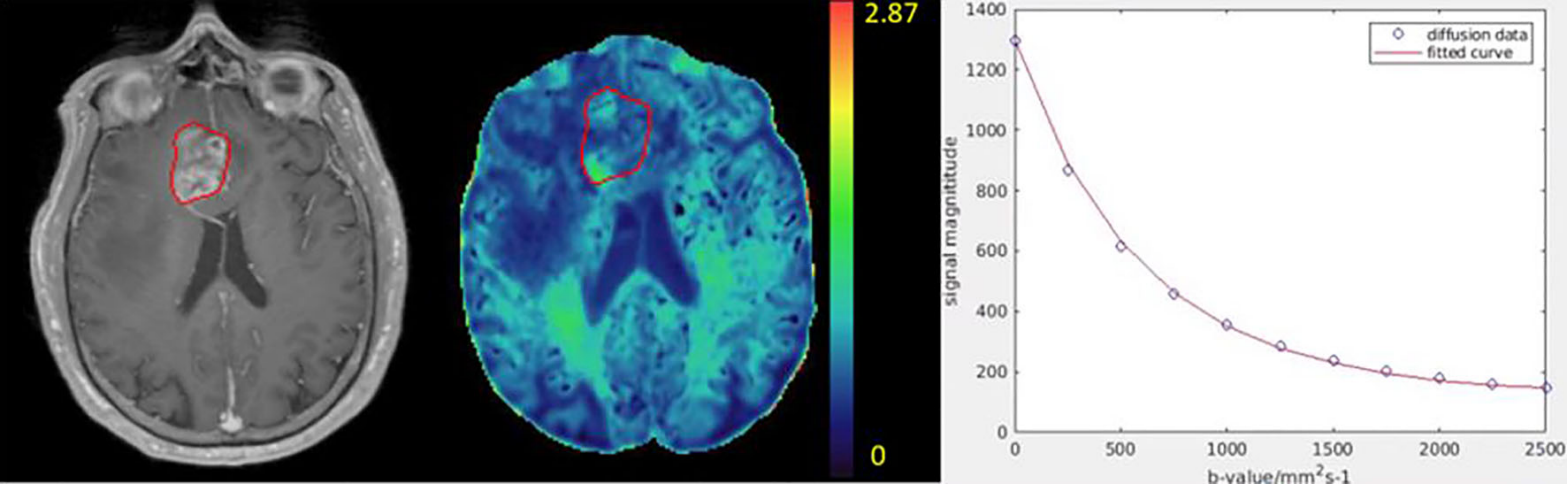
Illustration of a kurtosis map (color-coded, middle) of a patient with GBM. The color bar indicates kurtosis values. The post-Gd tumor volume (Gd-GTV, red contour) delineated on T1-weighted images (left) is overlaid on the kurtosis map. An example of diffusion weighted signals fitted by the diffusion kurtosis model is shown in the right panel. Blue dots represent original diffusion signal data in the Gd-GTV, and red solid line is the fitted curve. Note that the diffusion kurtosis model fits the diffusion signals well.

Kurtosis and DC maps of the 33 patients pre-RT and mid-RT were calculated. An example of kurtosis maps and the diffusion curve in the Gd-GTV is shown in **Figure 2**. Note that the kurtosis values in the Gd-GTV were heterogeneous. We investigated the mean kurtosis values in the Gd-GTV as well as the 80 and 90 percentile values pre-RT and mid-RT. Similarly, we investigated the mean DC, 10 and 20 percentile values in the Gd-GTV pre-RT and mid-RT. All data are summarized in **Table 2**.

**Table 2:** Kurtosis and DC values in the Gd-GTV pre-RT, mid-RT and post-RT.

	Pre-RT	Mid-RT	Post-RT
Mean Kurtosis ± SD	0.76 ± 0.10	0.73 ± 0.18	0.65 ± 0.14
80 percentile Kurtosis ± SD	1.07 ± 0.18	0.98 ± 0.27	1.04 ± 0.67
90 percentile Kurtosis ± SD	1.18 ± 0.24	1.07 ± 0.31	1.23 ± 1.04
Mean DC (um^2^/ms) ± SD	1.54 ± 0.30	1.67 ± 0.34	1.71 ± 0.43
10 percentile DC (um^2^/ms) ± SD	0.89 ± 0.13	1.07 ± 0.17	1.81 ± 0.47
20 percentile DC(um^2^/ms) ± SD	1.02 ± 0.15	1.20 ± 0.20	1.89 ± 0.51

**Figure 3:**
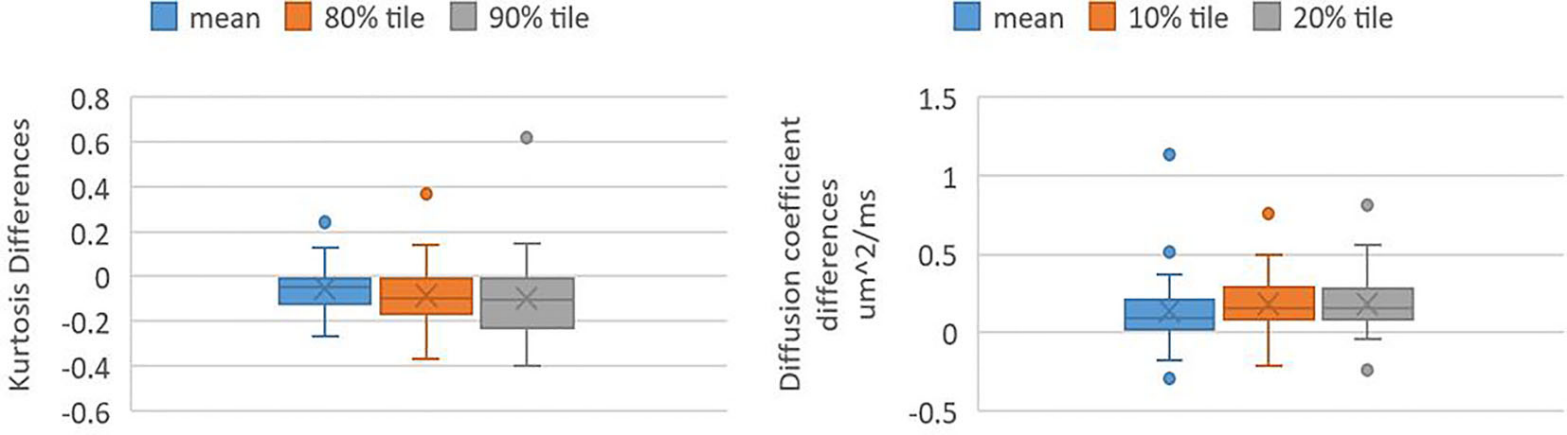
Box and whisker plots shows values of kurtosis differences and DC differences in Gd-GTV pre-RT and mid-RT (mid-RT values–pre-RT values). Left panel shows kurtosis differences of mean, 80 and 90 percentile kurtosis values. Right panel shows DC differences of mean, 10 and 20 percentile DC values.

The kurtosis values and DC values in the Gd-GTV at mid-RT decreased and increased significantly (P-value <0.005) compared to pre-RT, respectively (**Figure 3**). The three outlier data points in the kurtosis plot that did not follow the decrease group trends from pre-RT to mid-RT were from one patient who had rapid progression after treatment. In the DC plot, five outliers that deviated from the group trend came from three patients and were due to necrosis, tumor infiltration in the ventricle or adjacent to the surgical cavity.

The post-RT mean kurtosis, 80 and 90 percentile values, and mean DC, 10 and 20 percentile values in the Gd-GTV of the 21 patients are summarized in **Table 2**. Note that the post-RT mean kurtosis and DC values continued decreasing and increasing from the values from mid-RT, respectively. The large variances of kurtosis and DC over the group could be due to progression observed in two patients at 3 months post-RT.

### Correlation of Parameters With OS and PFS

Univariate Cox model analysis showed that large mean, 80 and 90 percentile kurtosis values in the Gd-GTV pre-RT were significantly associated with reduced OS (respective HR = 2.10, p = 0.03; HR = 2.29, p = 0.03; and HR = 2.30, p = 0.03; **Table 4**), but not the values measured at mid-RT (p-value >0.83) and post-RT (p-value >0.47). The DC values including the mean, 10 and 20 percentile from the Gd-GTV pre-RT, mid-RT and post-RT did not show any significant association with OS (p-value >0.3). Univariate Cox model analysis of clinical prognostic factors and dose for prediction of OS are listed in **Table 3**.

**Table 3:** Univariate Cox model analysis of DKI parameters and clinical factors for prediction of OS.

Parameters	Hazard ratio (HR)	p-value	95% CI
Mean K pre-RT	2.10	0.03*	[1.10, 4.02]
80 percentile K pre-RT	2.29	0.03*	[1.10, 4.71]
90 percentile K pre-RT	2.30	0.03*	[1.07, 4.96]
Gd-GTV pre-RT	0.74	0.25	[0.44, 1.23]
Age	1.72	0.14	[0.84, 3.52]
MGMT	0.45	0.2	[0.14, 1.47]
Dose	1.20	0.07	[0.98, 1.46]
EOR	0.34	0.52	[0.63,2.52]

*Significant with p < 0.05. The continuous data were normalized.

**Table 4:** Multivariate cox model analysis of clinical factors and MK for prediction of OS.

parameters	Hazard ratio (HR)	p-value	95% CI
Age	2.92	0.03*	[1.08, 7.94]
MGMT	0.25	0.09	[0.05, 1.24]
EOR	0.55	0.21	[0.21, 1.42]
mean K pre-RT	3.06	0.009*	[1.32,7.13]

*Significant with p < 0.05. The continuous variables were normalized to their means and standard deviations.

We further addressed the question whether kurtosis values in the Gd-GTV added any values than clinical prognostic factors, such as EOR, age, MGMT and Gd-GTV, for prediction of OS. Due to the similarity that exists among mean, 80 and 90-percentile kurtosis values in the Gd-GTV, we only selected the mean kurtosis in the analysis. After adjusting these clinical factors, the mean kurtosis value pre-RT was a significant predictor of OS (HR = 3.06, p < 0.009), see **Table 4**.

The mean, 80 and 90 percentile kurtosis values and the DC values in the Gd-GTV pre-RT and mid-RT were not significant predictors for PFS (p >0.5) using univariate Cox model analysis. However, the post-RT values of kurtosis, specifically, the large values of kurtosis at the 80 and 90 percentile in the Gd-GTV were associated with reduced PFS (p = 0.05) in the univariate Cox model analysis (p = 0.03 and p = 0.05, respectively), see **Table 5**, which could be a useful indicator for time of progression.

**Table 5:** Univariate Cox model analysis of DKI parameters post-RT for prediction of PFS.

Parameters	Hazard ratio (HR)	p-value	95% CI
Mean K post-RT	1.85	0.10	[0.88, 3.88]
80-percentile K post-RT	2.18	0.03*	[1.10, 4.30]
90-percentile K post-RT	1.82	0.05*	[1.00, 3.33]

*Significant with p < 0.05. The continuous data were normalized.

## Discussion

In this study, we investigated the diffusion kurtosis model and characterized non-Gaussian diffusion properties in the Gd-GTVs pre-RT, mid-RT and post-RT in the patients with GBM. We found that the mean kurtosis value in the Gd-GTV pre-RT was significantly prognostic of OS as a high mean kurtosis was associated with inferior of survival. Also, the diffusion kurtosis added a predictive value to the extent of surgery, age and methylation status for survival. The post-RT kurtosis values in the Gd-GTV predicted time to progression. In addition to glioma grading (14, 17, 20), the kurtosis model has potential to aid in conventional MRI for outcome prognosis. Further validation with another cohort of patients will warrant the role of the diffusion kurtosis model in the clinical management of GBM.

Many diffusion models have been investigated in gliomas. An apparent diffusion coefficient quantified from conventional DW images with b-values between 0 and 1,000 s/mm^2^ using a mono-exponential decay is the commonly reported parameter in literature. Previous studies have suggested that a low ADC was associated with a decrease in survival for patients with gliomas (23–27). One limitation of the mono-exponential model is that there are large deviations of fitted curves from the diffusion weighted signals with b-values greater than 1,500 s/mm^2^. Another problem is that with a single diffusion parameter is hard to describe the complex microstructure effects on water diffusion. To deal with the deviation of diffusion weighted signals from the mono-exponential function, a bi-exponential model with fast and slow diffusion components has been proposed (11). In the initial interpretation of the bi-exponential model, fast and slow diffusion coefficients are considered from respective extra- and intra-cellular water compartments, but the estimated fraction of the intra-cellular water in the tissue from the bi-exponential model cannot be matched with that measured by other methods (9). The bi-exponential model fits the diffusion curves better than the mono-exponential model. A study suggests that the fast diffusion coefficient is close to the reported human brain diffusion coefficient (28). To fit the bi-exponential model, it is necessary to take diffusion weighted images with more b-values, which increases the acquisition time. In addition, the bi-exponential model that fits four parameters is unstable to noise, which makes it difficult to generate high quality voxel-by-voxel brain maps.

In addition to the mono and bi-exponential models, other high order diffusion models that have been investigated in clinical gliomas, such as the fractional order calculus model (FROC) and restricted diffusion model (RDM) (12, 18). Those high-order diffusion models require diffusion weighted images with more b-values and high SNR. The FROC requires b-values up to 4,000 s/mm^2^, and the diffusion coefficient in the model is pre-determined by fitting a mono-exponential model before fitting the entire model (12), which may lead to some errors in parameters. The RDM is insensitive to intracellular diffusion coefficient and is instable to voxel fitting (18), which leads to difficulty in generating parameter maps in the patients with GBM. The diffusion kurtosis model improves the goodness of fit and is more stable than those high-order diffusion models (12, 18). In addition, the kurtosis model has been investigated in clinical gliomas (20). Research suggests that mean kurtosis shows better separation of glioma grades than fractional anisotropy and mean diffusivity. Overall, the kurtosis model is convenient to generate voxel maps and provides the potential measurement of non-Gaussian diffusion in GBM.

When considering underlying of tissue morphology and physiology of diffusion parameters, low ADC is considered to correlate with high cellularity. However, co-existence of edema and high vascularity in a single pixel of the tumor can elevate ADC compared to normal white matter and gray matter. To mitigate the influence of perfusion on measured diffusion coefficients, a bi-exponential model that quantifies fast and slow DCs has been investigated. The fast DC derived from the model is found to be significantly higher in high-grade gliomas than in low-grade gliomas (20), which could be due to hyper-vascularization in the high-grade gliomas. One limitation of the bi-exponential model is that the fraction of slow DC component has discordance with microstructure parameters, *e.g.*, the fraction of intra-cellular water. Some investigations suggest that the discordance may result from the restricted cell membrane and cell size (29, 30). The RDM considers restricted intracellular diffusion and modulations of diffusion gradients into the model (16, 18). To obtain accurate estimations of the apparent cell radius and the extracellular diffusion coefficient derived from the RDM in the GBM and brain normal tissue requires short diffusion times that may be beyond the clinical scanner hardware. The heterogeneous tissue could present even more challenges for the model (18). The FROC model shows that DC, fractional order and spatial parameter all differentiate high-grade pediatric brain tumors from low-grade ones (12). In addition, the fractional order has high predictive values for tumor outcomes (12). There are also some limitations of the FROC model. First, the parameters derived from the model may not differentiate tumor from normal tissue (12). Another challenge is that parameters are not sensitive enough to generate high contrast maps (12).

Previous research has suggested that the mean kurtosis could serve as the optimal parameter for grading glioma in practice (20). Zhang et al. investigated the correlation between OS and kurtosis in high grade gliomas, including grade III and grade VI, and found that mean kurtosis of glioma was a significant predictor of OS (21). Hempel et al. also assessed whether mean kurtosis was a prognostic factor in grade II, grade III, and grade IV gliomas, and found PFS and OS were significantly better in patients with lower mean kurtosis (31). However, different grades of gliomas could have specific features, which may contribute to prediction power. In our analysis, we only included grade IV glioma.

In this study, we found that high mean kurtosis values pre-RT were significantly correlated with reduced OS. To illustrate the unique contribution of the mean kurtosis, we also tested the Gd-GTV for prediction of survival using the cox model. We found that the Gd-GTV volume itself did not predict OS, which suggests that the mean kurtosis provides information beyond the enhanced tumor volume. In the Gd-GTV that consists of heterogeneous tumor with mixture of high cellular tumor and edema, the kurtosis values vary from high to low. The region with high kurtosis values may imply an aggressive component in the tumor, which is supported by the observations: 1) a higher grade of gliomas associated with higher mean kurtosis values (17, 20) and 2) high mean kurtosis values in GBM associated with inferior survival. The decreased mean kurtosis and the increased DC in the Gd-GTV of GBM after receiving radiation treatment are expected to represent a tumor response to therapy, but not specific enough to predict outcomes. Radiation likely causes cell degeneration and necrosis (32), which may decrease mean kurtosis and increase DC to an extent for some GBMs. In contrast, we observed substantially increased mean kurtosis and decreased DC at the mid-RT in two patients who had rapid tumor growth. Further research could be carried out to investigate pathology associated with mean kurtosis changes.

## Limitations

The DKI model quantifies non-Gaussian water diffusion in heterogeneous tissue and demonstrates the potential to predict OS in GBM patients. However, there are some limitations in the current study. First, we used 11 b-values up to 2,500 s/mm^2^, which increase acquisition time. Also, the model is sensitive to noise. To overcome the noise influence on model fitting, we applied a 2D Gaussian filter that blurs images. Second, the mean kurtosis decreased while the DC increased in mid treatment, but these changes are not significantly associated with survival. This may be affected by radiation treatment or the small patient sample size. Third, this is a retrospective analysis with a small sample size. Fourth, pathology correlated to the imaging finding is lacking in our research. Understanding of the mean kurtosis and DC changes after radiation and relationship to tumor changes is limited. The DKI model needs to be further validated in an independent large cohort of patients in future.

## Conclusions

The DKI model demonstrates the potential to predict OS and PFS in the patients with GBM. The model needs to be further investigated with pathologic correlation and validated in an independent large cohort of patients in the future. Further development and histopathological validation of the DKI model will warrant its role in clinical management of GBM.

## Data Availability Statement

The original contributions presented in the study are included in the article/supplementary material. Further inquiries can be directed to the corresponding author.

## Ethics Statement

The studies involving human participants were reviewed and approved by IRB. The patients/participants provided their written informed consent to participate in this study.

## Author Contributions

YC contributed to conception and design of the study. MK, DW, and HP helped collect data. YL performed the imaging analysis and statistical analysis. YL also wrote the first draft of the manuscript. YC, MK, and TL revised the manuscript. All authors contributed to manuscript revision, read, and approved the submitted version.

## Funding

This work was in part supported by NIH/NCI grant UO1 CA183848.

## Conflict of Interest

The authors declare that the research was conducted in the absence of any commercial or financial relationships that could be construed as a potential conflict of interest.
